# NOX-induced oxidative stress is a primary trigger of major neurodegenerative disorders

**DOI:** 10.1016/j.pneurobio.2023.102539

**Published:** 2023-10-12

**Authors:** Yuri Zilberter, Dennis R. Tabuena, Misha Zilberter

**Affiliations:** a Aix-Marseille Université, INSERM UMR1106, Institut de Neurosciences des Systèmes, Marseille, France; b Gladstone Institute of Neurological Disease, San Francisco, CA, USA

**Keywords:** Neurodegeneration, Oxidative stress, NADPH oxidase, Glucose hypometabolism, Alzheimer’s disease, Parkinson’s disease

## Abstract

Neurodegenerative diseases (NDDs) causing cognitive impairment and dementia are difficult to treat due to the lack of understanding of primary initiating factors. Meanwhile, major sporadic NDDs share many risk factors and exhibit similar pathologies in their early stages, indicating the existence of common initiation pathways. Glucose hypometabolism associated with oxidative stress is one such primary, early and shared pathology, and a likely major cause of detrimental disease-associated cascades; targeting this common pathology may therefore be an effective preventative strategy for most sporadic NDDs. However, its exact cause and trigger remain unclear. Recent research suggests that early oxidative stress caused by NADPH oxidase (NOX) activation is a shared initiating mechanism among major sporadic NDDs and could prove to be the long-sought ubiquitous NDD trigger. We focus on two major NDDs - Alzheimer’s disease (AD) and Parkinson’s disease (PD), as well as on acquired epilepsy which is an increasingly recognized comorbidity in NDDs. We also discuss available data suggesting the relevance of the proposed mechanisms to other NDDs. We delve into the commonalities among these NDDs in neuroinflammation and NOX involvement to identify potential therapeutic targets and gain a deeper understanding of the underlying causes of NDDs.

Neurodegenerative diseases (NDDs) resulting in cognitive impairment and dementia pose a significant therapeutic challenge, as effective treatment and prevention strategies remain elusive despite decades of intensive research. One of the main reasons for this is the lack of understanding of the primary initiating factors of sporadic NDDs, and therefore discovering these factors is crucial to developing a cure. Recent research suggests that there is one specific initiating mechanism that is shared among major sporadic NDDs which could prove to be just such a ubiquitous NDD trigger: early oxidative stress caused by NADPH oxidase (NOX) activation.

Major sporadic NDDs share many risk factors (Armstrong, 2020) and exhibit similar pathologies in their early stages (Chen et al., 2022; Lanznaster et al., 2022; Teleanu et al., 2022), indicating the existence of common initiation pathways. Glucose hypometabolism with associated oxidative stress are early biomarkers of most major NDDs (Butterfield et al., 2022; Liu et al., 2017; Tang, 2020; Zilberter and Zilberter, 2017) and likely a major cause of detrimental disease-associated cascades. Treating early glucose hypometabolism and oxidative stress may therefore be an effective preventative strategy for most sporadic NDDs. However, the exact trigger and source of this onset pathology remain critical questions. Emerging evidence indicates that initiating oxidative stress (“iOS”) inhibits brain glucose metabolism during NDD prodromal stages, making iOS a major pathogenic factor rather than just an early symptom. We argue that iOS is largely the result of NOX hyperactivation, making this unique enzyme a potential primary trigger of NDDs (Begum et al., 2022; Malkov et al., 2021; Tarafdar and Pula, 2018; Vermot et al., 2021; Waghela et al., 2021) and a promising target for preventive treatment development. Furthermore, iOS stimulates the misfolding and aggregation of key NDD-associated proteins such as beta-amyloid (Aβ), tau, or alpha-synuclein (Abramov et al., 2020; Cheignon et al., 2018; Piccirillo et al., 2022; Singh et al., 2019). These pathogenic peptides in turn promote oxidative stress through NOX activation (Abramov et al., 2020; Piccirillo et al., 2022; Singh et al., 2019), thus establishing and driving the vicious cycle of NDD pathogenesis.

In this review paper, we will extensively focus on the major NDDs that afflict millions worldwide. Alzheimer’s disease (AD) is the most prevalent form of dementia, accounting for 60%– 80% of all dementia cases, and will be the primary focus of this review. Parkinson’s disease (PD), which is the second most common neurodegenerative disorder (Erkkinen et al., 2018) will also be discussed, along with frontotemporal dementia (FTD), a tauopathy (Yoshiyama et al., 2001; Y. Zhang et al., 2022) accounting for 10–20% of dementia cases and the second most common form of dementia in people under 65 years (Young et al., 2018). We will also delve into acquired epilepsy, which has been recognized as a risk factor for both AD (Tombini et al., 2021; D. Zhang et al., 2022) and PD (Cano et al., 2021; Gaitatzis et al., 2012; Schrag et al., 2022) as well as a comorbidity of both diseases due to the presence of brain hyperactivity and seizures (Cano et al., 2021; Negi et al., 2023; Neri et al., 2022). We will discuss the data from our recent studies on causes and functional consequences of NOX activation in AD and acquired epilepsy models, and how they contribute to the general understanding of NOX involvement in neurodegenerative processes applicable to all NDDs. Finally, we will briefly discuss Amyotrophic Lateral Sclerosis (ALS) and multiple sclerosis (MS), both commonly associated with dementia (Benedict et al., 2020; Geser et al., 2009; Londoño et al., 2022) as 50% of ALS cases co-present with FTD (Ling et al., 2013; Lomen-Hoerth et al., 2003; Zago et al., 2022). By exploring all these NDDs and their commonalities in oxidative stress and NOX involvement, we aim to identify potential therapeutic targets and gain a deeper understanding of the underlying causes of NDDs. To achieve this, we will undertake a comprehensive analysis of existing literature, with the hope of contributing to the growing body of research on NDDs and paving the way for future breakthroughs in the field.

## 1. Glucose hypometabolism in NDDs

We have previously reviewed the available literature on glucose hypometabolism and its key role in pathogenesis of AD, PD, and acquired epilepsy (Zilberter and Zilberter, 2017), updated in a recent comprehensive review by Butterfield et al. (Butterfield et al., 2022). Regarding the other NDDs, progressive and spreading glucose hypometabolism is commonly observed in FTD, with divergent patterns across subtypes that can discriminate the disease from AD cases (Garrett and Niccoli, 2022; McKenna et al., 2023). Importantly, longitudinal studies on familial FTD cases demonstrated that glucose hypometabolism precedes disease symptoms (Arvanitakis et al., 2007; Clarke et al., 2021; Deters et al., 2014; De Vocht et al., 2020; Jacova et al., 2013; McKenna et al., 2023), appearing 7–12 years prior to disease onset (Clarke et al., 2021; Jacova et al., 2013). ALS is also associated with glucose hypometabolism (Blasco et al., 2020; Tefera et al., 2021; Tefera and Borges, 2016), which has been suggested to precede neurodegeneration and symptoms (Beal, 1992; Browne et al., 2006; So et al., 2018). Likewise, altered energy metabolism has been linked to MS initiation and progression (Mathur et al., 2014; Papiri et al., 2023), with mitochondrial injury a potential major driver of tissue damage in MS (Dutta et al., 2006; Lu et al., 2000; Mahad et al., 2008).

It has been presumed that hypometabolism in NDDs might be secondary to brain atrophy and neuronal loss. Indeed, NDDs are characterized by a progressive loss of specific neural clusters contributing to the unique pattern of functional and cognitive deficits in each disease. The exact pathophysiological mechanisms underlying neuronal loss are poorly understood, although energy deficiency at different neural hierarchies was hypothesized as a root cause (Muddapu et al., 2020). The FDG-PET technique normally used for metabolic measurements in humans lacks the resolution to measure glucose uptake at a cellular level and thus cannot determine which cells contribute to the observed glucose hypometabolism.

Local cortical atrophy associated with hypometabolism is typically detected in diagnosed AD (e.g., (Croteau et al., 2018; Strom et al., 2022)) and PD (Borghammer, 2012; Hall and Lewis, 2019; Yu et al., 2020) patients. This correlation, however, is much less evident in the early or prodromal stages of the disease. A lower association between gray matter volume reduction and hypometabolism in mild cognitive impairment (MCI) patients was reported (Wirth et al., 2018), while another recent study found no such relationship (Li et al., 2023), and yet another reported hypometabolism without any atrophy at all (Kljajevic et al., 2014). Croteau et al. (Croteau et al., 2018) found a 93% reduction of glucose hypometabolism in the cingulate gyrus of MCI patients compared to healthy controls, while gray matter volume reduction in the same patients was confined to the temporal cortex. Assessing glucose metabolism based on distinct brain regions, as opposed to the unit of brain tissue, yielded valuable insights into how the extent of brain atrophy influences glucose utilization. This approach revealed a remarkable regionalization of glucose hypometabolism, in contrast to the more diffuse nature of the widespread structural changes, indicating that the two pathologies might not be as tightly linked as previously assumed.

In addition, increasing evidence suggests that changes in energy metabolism occur prior to significant brain atrophy and the onset of clinical symptoms. Several human studies revealed that despite brain glucose hypometabolism, regional brain atrophy, and cortical thinning in MCI and early AD patients, brain ketone uptake in the same brain regions remained similar to that of healthy controls (Castellano et al., 2015; Croteau et al., 2018; Cunnane et al., 2020), further confirming that the AD-associated glucose hypometabolism is due to the reduction in glucose utilization rather than any structural pathologies.

Because cerebral blood flow is an essential source of glucose supply to neural tissue, disruptions in cerebrovascular function may also result in brain metabolic deficiency. Reduced cerebral blood flow was reported in multiple studies of AD (Apátiga-Pérez et al., 2022; Solis et al., 2020), as well as other NDDs including PD (Al-Bachari et al., 2017; Apátiga-Pérez et al., 2022; Borghammer, 2012; Borghammer et al., 2010; Derejko et al., 2001; Shang et al., 2021), FTD (Dopper et al., 2016), HD (Drouin-Ouellet et al., 2015), and ALS (Kew et al., 1993). However, results of a recent study on AD patients using a unique combination of imaging and cerebrospinal fluid (CSF) measures (Ahmadi et al., 2023) suggest that hypoperfusion might not be an early event in the preclinical phase of the disease and is therefore unlikely to significantly contribute to glucose hypometabolism in AD.

Cerebral blood flow in PD patients was thoroughly investigated in many studies (see comprehensive analysis in Borghammer, 2012). Early-stage PD patients displayed clusters of hypoperfusion in medial occipital lobe, precuneus, and lateral prefrontal cortex despite widespread cortical hypometabolism (Berti et al., 2012; Borghammer, 2012; Borghammer et al., 2010). It was noted that although some amount of cortical hypometabolism could be explained by atrophy, this hardly accounts for the cortical hypometabolism in its entirety as many studies of early-stage PD patients reported little or no atrophy (Borghammer et al., 2010). Importantly, analysis of more than 30 studies revealed that the resting state in PD patients is characterized by various degrees of hypoperfusion and hypometabolism in cerebral cortical structures, but no confirmed hypermetabolism/hyperperfusion was found in the same regions (although possible hypermetabolism was suspected in some subcortical regions, e.g., external pallidum) (Borghammer, 2012; Borghammer et al., 2012). Later studies of idiopathic PD patients revealed no significant variations in whole-brain CBF but found small regions of lower perfusion (Al-Bachari et al., 2017) (see also (Shang et al., 2021)). Likewise, glucose hypometabolism was found to spread beyond hypoperfused areas in FTD patients (Anazodo et al., 2018), indicating a lack of causal relationship.

In line with the results from imaging studies, metabolomic blood/CSF signatures of disrupted energy metabolism have also been reported in multiple human studies of AD and PD (for review see (Maszka et al., 2023), FTD (Murley et al., 2020), ALS (Li et al., 2022), and MS (Porter et al., 2020), further confirming that NDD-related early brain hypometabolism is generally due to changes in glucose utilization rather than to structural degeneration.

Finally, insulin resistance is both a risk factor and an associated pathology of all major NDDs including AD (Bloom et al., 2018; Kandimalla et al., 2017), FTD (Ahmed et al., 2014), PD (De Pablo-Fernandez et al., 2018; Hogg et al., 2018; Pagano et al., 2018; Sandyk, 1993; Sharma et al., 2021; Xu et al., 2011), HD (Farrer, 1985), and ALS (Reyes et al., 1984; Sun et al., 2015). While insulin mediates peripheral glucose utilization, the uptake of glucose in the brain, traditionally believed to be independent of insulin (Havrankova et al., 1979, 1978), presents a more nuanced scenario. Neurons primarily rely on GLUT3, while astrocytes utilize GLUT1 (Koepsell, 2020), both of which are insulin-independent. However, recent studies point to involvement of insulin-dependent neuronal GLUT4 in cognitive function (Ashrafi et al., 2017; McEwen and Reagan, 2004; McNay and Pearson-Leary, 2020; Pearson-Leary and McNay, 2016), as well as of insulin and IGF-1 signaling in astrocytic glucose transport through mediating GLUT1 translocation to the membrane (Fernandez et al., 2017; Hernandez-Garzón et al., 2016). Moreover, peripheral insulin resistance was shown to correlate with reduced brain glucose utilization in MCI and AD patients (Willette et al., 2015). Consequently, disruptions in insulin signaling pathways might indeed contribute to the observed glucose hypometabolism in the pathogenesis of NDDs.

In summary, while we cannot entirely discount the potential contributions of pathological events like neuronal atrophy and hypoperfusion, current evidence indicates that glucose hypometabolism during the early stages of NDDs predominantly arises from impaired glucose uptake and utilization at the cellular and molecular levels, reflecting dysfunction of the brain’s energy metabolism pathways.

## 2. NDD-associated glucose hypometabolism is likely initiated by oxidative stress

The excess ROS can damage cellular lipids, proteins, or DNA inhibiting their normal function. Because of this, oxidative stress has been implicated in a number of human diseases as well as in the aging process (Jomova et al., 2023; Ushio-Fukai et al., 2021). In particular, ROS inhibit critical glycolytic enzymes such as glyceraldehyde 3-phosphate dehydrogenase (GAPDH) (Peralta et al., 2015) and pyruvate kinase M2 (PKM2) (Anastasiou et al., 2011), reducing the glycolytic rate. In addition, ROS also induce a variety of post-translational protein modifications, including cysteine oxidation in the form of sulfenylation and S-glutathionylation. These modifications can directly influence the activity of susceptible metabolic pathways (Holmström and Finkel, 2014; Sies and Jones, 2020). It is important to note that reactive nitrogen species (RNS), which we do not consider in this review, can also contribute significantly to the eventual brain oxidative stress (e.g., reviewed in (Cobb and Cole, 2015; Singh et al., 2019)).

**Fig. 1.**
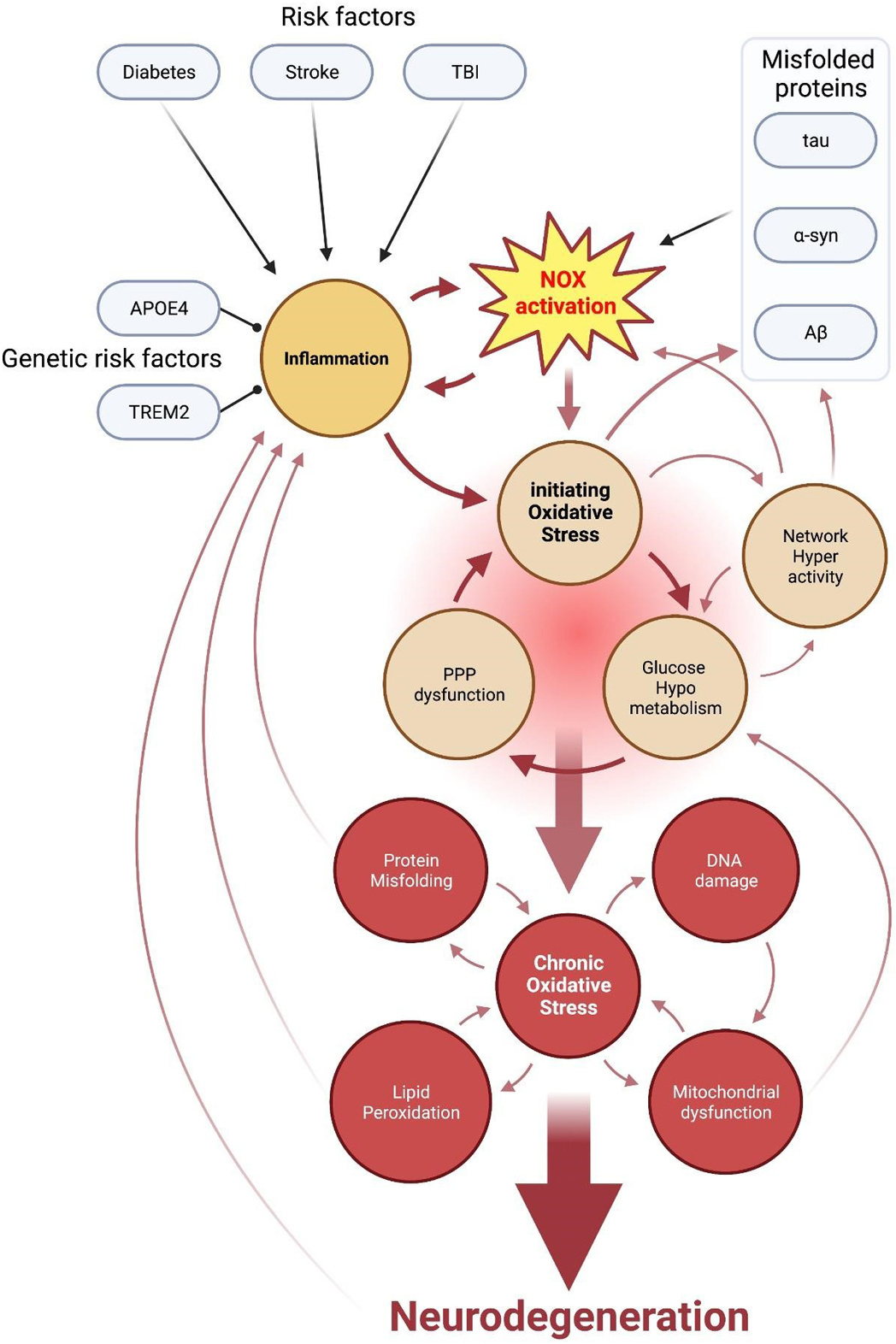
The common pathway of neurodegenerative disease initiation and progression. Multiple shared risk factors such as diabetes, stroke, and traumatic brain injury (TBI) trigger the NOX activation and neuroinflammation cycle exacerbated by major genetic risk factors such as APOE4 and TREM2. In addition, diabetes-related hyperglycemia as well as various NDD-associated endogenous misfolded proteins trigger NOX activation. The NOX-inflammation loop results in initiating oxidative stress (iOS) that impairs glucose metabolism and leads to a disrupted pentose-phosphate pathway (PPP), disabling the primary cytosolic antioxidative defense system and perpetuating the vicious cycle of oxidative stress. NOX-mediated iOS also promotes protein misfolding and induces network hyperactivity. This further worsens the hypometabolism and boosts NOX activity as well as misfolded protein release. With time, these multiple pathological cycles induce chronic oxidative stress that perpetuates multiple neurodegenerative processes such as lipid peroxidation, protein misfolding, DNA damage and mitochondrial dysfunction, all feeding back into the initial pathologies, and which ultimately results in tissue damage and neurodegeneration.

To maintain a balanced interplay between the generation and neutralization of ROS and to counteract oxidative stress, brain cells rely on various antioxidant systems. Among these, the pentose-phosphate pathway (PPP) stands out for its efficiency and rapid response times (TeSlaa et al., 2023). PPP starts with glucose 6-phosphate, which also feeds glycolysis, and comprises two branches with the oxidative PPP producing cellular NADPH that is required for neutralization of H_2_O_2_ by the glutathione system, and the non-oxidative PPP producing pentose (5-carbon) sugars (Cherkas et al., 2020; Dienel, 2019; Tang, 2019). The PPP’s role as an antioxidant defense mechanism represents one of the swiftest adaptive responses of brain cells to sudden oxidative stress. The glutathione system activation in response to oxidative insults is nearly immediate, whereas the transcriptional response takes considerably longer (Cherkas et al., 2020; Stincone et al., 2015). PPP’s contribution constitutes approximately 3–7% of total brain glucose utilization in adult animals (Dienel, 2019; Sies and Jones, 2020; Winterbourn, 2018) and about 7% in healthy humans (Dusick et al., 2007), but can reach up to 30% during acute oxidative stress (Cherkas et al., 2020; Dienel, 2019; Stincone et al., 2015). Therefore, PPP has a large reserve capacity for rapid upregulation, ensuring tight control of ROS levels even during physiological NOX activation. However, even PPP may be unable to cope with pathological ROS accumulation resulting in the establishment of initiating oxidative stress. Chronic glucose hypometabolism induced by iOS is in turn likely to result in further amplification of oxidative stress since glucose is the sole fuel of the PPP (Cherkas et al., 2020; Dienel, 2019; Tang, 2019). This creates a positive feedback cycle between oxidative stress and glucose hypometabolism which promotes neurodegenerative cascades of NDD pathogenesis (Fig. 1).

On the slower time scale, the cellular redox state is also controlled by specific gene transcription factors. Nuclear factor erythroid 2 (NF-E2)-related factor 2 (Nrf2) is a key transcription factor that coordinates the cellular antioxidant response by regulating the expression of oxidative stress-related genes enclosing the antioxidant response element (ARE) in their promoters. During oxidative stress, Nrf2 migrates to the nucleus and binds to ARE, activating the transcription of genes responsible for the induction of enzymes involved in antioxidant defense (Tonelli et al., 2018; Vardar Acar and Özgül, 2023). The Nrf2 system plays a central role in protecting cells from environmental stressors and also suppresses pathological inflammation. Dysfunctions in the Nrf2/ARE signaling pathway result in oxidative stress and are implicated in NDD progression (Uruno and Yamamoto, 2023; Zgorzynska et al., 2021). Due to its protective properties, Nrf2 activation has been suggested to be an effective therapy strategy for multiple NDDs (Amoroso et al., 2023).

There is widespread agreement that oxidative stress is a key contributor to the pathogenesis and progression of AD (Rummel and Butterfield, 2022). Oxidative damage has been suggested to be a major contributor to inefficient glucose utilization in AD (Butterfield, 2023; Butterfield and Halliwell, 2019). Proteomics studies in early AD patients identified Aβ_1–42_ -mediated oxidative modifications to glycolytic enzymes (Butterfield and Boyd-Kimball, 2019). As glucose metabolism is significantly depressed in the brains of patients diagnosed with MCI, several glycolytic and mitochondrial proteins are dysfunctional due to oxidative modification and likely contribute to the deficient glucose utilization in AD and MCI brains. In the 3xTg-AD murine model, oxidative stress resulted in brain insulin resistance which may further contribute to impaired glucose metabolism, BBB dysfunction, and energy supply shortage (Butterfield et al., 2022).

Oxidative stress is a also well-established driver of the PD pathogenesis, with numerous studies reporting evidence of oxidized DNA, lipids, and proteins in the brain tissues of both familial and sporadic PD patients (Aborode et al., 2022; Chang and Chen, 2020; Deas et al., 2016; Dorszewska et al., 2021; Puspita et al., 2017). In vitro studies using induced pluripotent stem cell-derived neurons obtained from PD patients (Devine et al., 2011; Zhang et al., 2004) have further demonstrated that oligomeric α-synuclein generates ROS in these cells, independent of mitochondrial activity (Abramov et al., 2020; Deas et al., 2016). Among the etiological factors of sporadic PD, the impairment of the Nrf2/ARE pathway has been implicated (Brandes and Gray, 2020; Zgorzynska et al., 2021). Many studies indicate increased markers of oxidative damage along with decreased antioxidant enzyme activity in the brain and blood of PD patients (Khan and Ali, 2018; Wei et al., 2018). Decreased levels of G6PD, a rate-limiting enzyme of PPP, have been identified in the putamen of patients with early-stage PD, suggesting suppressed PPP and lowered antioxidant capacity (Dai et al., 2023; Dunn et al., 2014). Conversely, overexpression of G6PD in the nigrostriatal system was shown to be protective in a neurotoxin-induced mouse PD model (Mejías et al., 2006). SNc dopaminergic neurons are particularly vulnerable to oxidative stress, placing these neurons at risk for degeneration, especially when glucose metabolism is impaired. Oxidative stress damages dopaminergic neurons in PD in various ways and may be a direct trigger of associated glucose hypometabolism (Dai et al., 2023). For example, glycolytic enzymes GAPDH, aldolase A, and enolase 1 have been shown to be oxidatively modified by the lipid peroxidation product 4-hydroxynonenal in PD brains (Gómez and Ferrer, 2009).

Oxidative stress is also associated with neurodegeneration in ALS. Multiple studies reported elevated oxidative stress markers in CSF from sporadic ALS patients, such as oxidative DNA damage markers 8-oxodG (Bogdanov et al., 2000; Murata et al., 2008), advanced oxidation protein products (Djordjevic et al., 2017; Siciliano et al., 2007) and lipid peroxidation product HNE (Simpson et al., 2004) which increased with disease progression. However, the exact causative role of oxidative stress in ALS pathogenesis is debated, and other studies found variable CSF oxidative stress markers. For example, Siciliano et al. (Siciliano et al., 2007) reported increased advanced oxidation protein product levels but concurrently measured HNE and nitrites levels were not statistically different from controls. Another study of 10 ALS patients found no difference in CSF levels of nitrosative stress marker 3-nitrotyrosine compared to 6 controls (Mendonça et al., 2011).

MS disease development is considered to be mediated by the interaction of genetic predisposition, environmental factors, and dysfunctional immune response. While there are no reliable biomarkers to predict or diagnose disease onset, oxidative stress is one common feature in the brains of MS patients (Gilgun-Sherki et al., 2004; Zhang et al., 2020), with CSF oxidative stress markers reported in multiple studies (Pasquali et al., 2015; Trentini et al., 2017; Wang et al., 2014).

Direct evidence from our multiple studies on acquired epilepsy and AD in animal models further supports our hypothesis that impaired glucose utilization is the result of pathological oxidative stress (Zilberter et al., 2022). We demonstrated that exogenous H_2_O_2_ leads to inhibition of network activity-driven glucose consumption in naïve brain slices (Malkov et al., 2018), replicating the Aβ_1–42_ effect we observed both in *ex vivo* and in vivo experiments (Malkov et al., 2021). Moreover, acute oxidative stress triggers network hyperactivity and seizures in healthy tissue, while inhibition of ROS production by NOX blockade results in a pronounced reduction of epileptiform activity in vivo (Malkov et al., 2019). Likewise, chronic reduction of brain glycolysis by intraventricular 2-DG injections induces network hyperactivity and seizures in initially healthy rats (Samokhina et al., 2020, 2017). Finally, we have shown that the activation of NOX by Aβ_1–42_ leads to oxidative stress resulting in brain glucose hypometabolism, network hyperactivity, and neuropsychiatric-like symptoms (Malkov et al., 2021), directly linking NOX-mediated oxidative stress to the onset and progression of AD. While the primary aim of these studies was to uncover the mechanisms underlying acquired epilepsy and AD pathogenesis, our findings reveal a deeper, more fundamental connection between NOX hyperactivation, oxidative stress, glucose hypometabolism, and network dysfunction, with potential relevance to a broader spectrum of NDDs.

Thus, experimental data demonstrates a major role of oxidative stress in inducing glucose hypometabolism and network dysfunction. The principal question then is what is the origin of such iOS? If ROS overproduction is due to mitochondrial dysfunction as is commonly assumed, prevention of ROS overgeneration is problematic. At least 11 sites of mitochondrial ROS production have been identified (Brand, 2016; Napolitano et al., 2021), and therefore the proposed direct inhibition of mitochondrial ROS production, as opposed to the use of ROS scavengers (Angelova and Abramov, 2018), is challenging. The use of mitochondrially-targeted antioxidants failed in multiple clinical trials (Angelova and Abramov, 2018; Ienco et al., 2011; Jurcau, 2021; Kim et al., 2015; Kumar and Singh, 2015; Liu et al., 2017; Perez Ortiz and Swerdlow, 2019; Singh et al., 2019; Wang et al., 2019), and we found that potent exogenous antioxidants failed to abate rapid ROS accumulation during network activity in brain slices (Malkov et al., 2018). However, mounting evidence indicates that the primary source of iOS during NDD onset is not mitochondria but rather activated NOX, as we discuss below.

## 3. Minor involvement of mitochondrial dysfunction in iOS and early glucose hypometabolism

To understand the underlying causes of glucose hypometabolism, it is crucial to determine whether mitochondrial dysfunction precedes or occurs in parallel with the decrease in glucose consumption, or if glycolysis impairment is instead induced by oxidative stress from some other source that precedes mitochondrial dysfunction. Mitochondria are generally accepted to be the primary source of ROS production (up to 90%) under normal physiological conditions in brain cells (Andreyev et al., 2015; Balaban et al., 2005), and this has led to the notion of mitochondria-based oxidative stress in many reports. However, although the brain is assumed to have a rather weak antioxidant defense (Cobley et al., 2018; Millichap et al., 2021; Patel, 2016; Singh et al., 2019), this does not apply to mitochondria which have a highly efficient antioxidant defense system consisting of several detoxifying enzymes such as glutathione and catalase (see details in (Andreyev et al., 2005; Munro and Pamenter, 2019; Starkov, 2008)) that neutralize ROS as soon as they are generated. ROS are produced at various sites in mitochondria, but most are generated as by-products (hyperoxide, O^-^_2_) of the electron transport chain during oxidative phosphorylation, with subsequent dismutation of $O_{2}^{-}$ to H_2_O_2_ by copper and zinc superoxide dismutases (Cu, Zn-SOD) in the intermembrane space and manganese SOD (Mn-SOD) in the matrix (Angelova and Abramov, 2018; Tirichen et al., 2021; Venditti et al., 2013). The rate of H_2_O_2_ removal is two to three times faster than mitochondrial H_2_O_2_ production (Munro and Pamenter, 2019; Starkov, 2008) and as such, the physiological emission of ROS from mitochondria is negligible and may serve a signaling function (Andreyev et al., 2020; Zarse and Ristow, 2021). Additionally, due to their robust scavenging capabilities, mitochondria can also neutralize cytoplasmic ROS and may even serve as a ROS sink (Andreyev et al., 2015; Napolitano et al., 2021; Starkov, 2008).

Mitochondria in the AD brain are susceptible to accumulating oxidative damage (Butterfield and Boyd-Kimball, 2020; Dewanjee et al., 2022; Mecocci et al., 1997, 1994). During NDD pathogenesis, chronic oxidative stress and glucose hypometabolism may eventually lead to mitochondrial impairment, resulting in overproduction of mitochondrial ROS and energy deprivation, as demonstrated in several studies (Jurcau, 2021; Millichap et al., 2021; Onyango et al., 2021; Wang et al., 2019). Although it is generally accepted that oxidative stress is the primary cause of mitochondrial dysfunction during NDDs, it is unlikely that the initial origin of such oxidative stress is the mitochondria themselves, given their powerful antioxidant defense system and high intrinsic resistance to acute oxidative stress (Gandhi and Abramov, 2012; Napolitano et al., 2021; B. D. Zhang et al., 2022; Y. Zhang et al., 2022; B. Zhang et al., 2022). Rather, it is more plausible that extra-mitochondrial ROS accumulation via the activity of other sources, such as NOX, is involved in the early stages of NDD onset. This oxidative stress may ultimately result in the damage or dysfunction of mitochondria, but its initial and primary targets are likely cytoplasmic processes such as glycolysis.

Although mitochondrial dysfunction is a hallmark feature of major sporadic NDDs (Federico et al., 2012; Ozgen et al., 2022), the timing of mitochondrial oxidative damage emergence in relation to other pathologies remains unclear. It is widely acknowledged that impaired energy metabolism and oxidative damage are central to the pathogenesis of NDDs. Interestingly, while glucose hypometabolism is one of the earliest features of AD, previous studies reported that the cerebral metabolic rate of oxygen is not altered or is changed disproportionately to the prominent decrease in glucose utilization (Hoyer, 2004, 1992; Hoyer et al., 1988). Unaltered oxygen utilization and normal CO_2_ production indicate that mitochondrial function remains intact at the onset of AD (Hoyer et al., 1988). Further evidence from early studies using the arterio-venous difference method showed that brain ketone uptake is still normal in moderately advanced AD (Lying-Tunell et al., 1981; Ogawa et al., 1996). As ketone catabolism is entirely mitochondrial, these findings indicate that oxidative phosphorylation may still be normal at AD onset. Recent studies using PET ketone tracer, 11 C-acetoacetate (AcAc), have confirmed that brain metabolism of ketones is unchanged in MCI and early AD (Castellano et al., 2015; Croteau et al., 2018; Cunnane et al., 2020, 2016; Hoyer et al., 1988; Kapogiannis and Avgerinos, 2020), further supporting the notion that mitochondrial oxidative phosphorylation remains relatively undamaged at AD onset. It therefore appears that brain hypometabolism in prodromal AD may be limited to glucose and glycolysis (Cunnane et al., 2016) and does not involve dysfunctional mitochondrial oxidative phosphorylation.

Animal studies have suggested that mitochondria are not the main source of ROS overproduction in AD models (Angelova and Abramov, 2018; Gandhi and Abramov, 2012) or during seizure activity (Kovac et al., 2017; T. Shekh-Ahmad et al., 2019). Likewise, we did not observe any decreases in oxygen consumption during epileptiform network hyperactivity (Malkov et al., 2018) or following application of Aβ_1–42_ (Malkov et al., 2021; Zilberter et al., 2013), indicating unaltered mitochondrial function. However, a significant reduction in glucose utilization was detected in all cases, suggesting that glycolysis impairment is a primary pathology that precedes or parallels mitochondrial dysfunction.

Moreover, mitochondrial-targeted antioxidant therapies have failed to exhibit evident benefits in multiple clinical trials, supporting the proposition of negligible mitochondrial contribution to the iOS in major CNS diseases (Angelova and Abramov, 2018; Ienco et al., 2011; Jurcau, 2021; Kim et al., 2015; Kumar and Singh, 2015; Liu et al., 2017; Perez Ortiz and Swerdlow, 2019; Singh et al., 2019; Wang et al., 2019).

## 4. A major contribution of NOX to iOS

NOX enzymes are responsible for the respiratory burst in phagocytes (Bedard and Krause, 2007) and have a unique biological function of generating ROS. NOX activation has also been shown to be critical to neuroinflammatory response by directly regulating microglial proliferation (Mander et al., 2006) and stimulating cytotoxic nitric oxide and cytokine release (Chéret et al., 2008). NOX are multi-subunit enzymes comprising membrane and cytosolic subunits. Under resting conditions, NOX is dormant, and the cytosolic components remain dispersed in the cytosol. However, upon activation which requires specific agonists (such as NMDAR stimulation in neurons (Minnella et al., 2018)), cytosolic components translocate to the membrane and assemble into the functioning complex (Rastogi et al., 2016). NOX-generated ROS have been reported to be a major source of oxidative stress in several neurodegenerative diseases, including AD and PD (Barua et al., 2019; Ma et al., 2017; Tarafdar and Pula, 2018; Waghela et al., 2021), as well as acquired epilepsy and stroke (Brennan-Minnella et al., 2015; Eastman et al., 2020; Kovac et al., 2017; Lin et al., 2020). Our experiments have shown that NOX activation initiates spontaneous seizure-like events in mouse brain slices and results in oxidative stress and long-lasting glucose hypometabolism, while NOX inhibition prevents these pathologies (Malkov et al., 2019). NDD-associated misfolded proteins such as Aβ, Tau, and α-synuclein can activate NOX (Abramov et al., 2020; Esteras et al., 2021; Keeney et al., 2022; Shelat et al., 2008; Tarafdar and Pula, 2018). Hyperglycemia in type 2 diabetes patients, who are at a heightened risk for major NDDs such as AD, PD, and FTD, leads to oxidative stress via NOX activation through the increased synthesis of diacylglycerol and activation of protein kinase C (Volpe et al., 2018).

NOX expression has been discovered in multiple brain cell types (Hernandes et al., 2022; Sorce and Krause, 2009), with NOX2 and NOX4 being the most prominent isoforms detected in neurons, microglia, and astrocytes (Hernandes et al., 2022; Hou et al., 2020; Sorce et al., 2017). Interestingly, while the neuroinflammation-associated NOX expression locus is traditionally attributed to microglia, emerging evidence, including our own research, points to neuronal NOX activity as critical to the neuroinflammatory cascades. We have recently demonstrated a key role of neuronal NOX2 in mediating Aβ metabolic and network toxicity (Malkov et al., 2021), while others have shown neuronal NOX to drive neurodegeneration in mouse PD (Belarbi et al., 2017; Keeney et al., 2022; Tu et al., 2023) and tauopathy (Luengo et al., 2022) models (see corresponding sections below for more detail).

### 4.1. Alzheimer’s disease and frontotemporal dementia

Numerous clinical studies have reported hyperactivated NOX in the cortex of patients with MCI, indicating NOX’s potential role in the prodromal stage of AD (Ansari and Scheff, 2011; Bruce-Keller et al., 2010; Fragoso-Morales et al., 2021). Aβ was reported to induce brain oxidative stress (Butterfield and Halliwell, 2019) largely via activation of NOX (Abramov et al., 2020; Simpson and Oliver, 2020; Tarafdar and Pula, 2018). Multiple studies of AD patients have reported a correlation between Aβ levels and NOX2 activity, further demonstrating the role of NOX in AD (Simpson and Oliver, 2020). Post-mortem analyses of AD patients’ cerebral cortices have shown that oxidative stress resulting from NOX2 activation plays a significant role in the development of AD (Hou et al., 2020; Ma et al., 2017; Rastogi et al., 2016; Sorce et al., 2017; Tarafdar and Pula, 2018). Notably, NOX activity has been found to have a robust negative correlation with cognitive status in humans (Ansari and Scheff, 2011). In animal experiments, Aβ has been shown to induce oxidative stress in astrocytes, microglia, and neurons via the activation of NOX (Abramov and Duchen, 2005; Rastogi et al., 2016; Tarafdar and Pula, 2018). Park and colleagues demonstrated NOX2’s potential importance in AD by finding no indications of chronic Aβ toxicity in Tg2576 mice overproducing human Aβ but lacking NOX2 (Park et al., 2008). Our recent study found that Aβ reduces brain glucose consumption and glycolysis, resulting in long-lasting network dysfunction and behavioral changes (Malkov et al., 2020). Critically, toxic Aβ effects were prevented by NOX2 inhibition and were absent in NOX2-KO mice, establishing NOX2 as the key enzyme mediating Aβ toxicity. NMDAR blockade by APV mimicked the preventative effects of NOX2 inhibition, suggesting that neuronal NOX2 (Minnella et al., 2018) is mainly responsible for the observed Aβ effects.

Cerebral hypoperfusion is implicated in AD and other NDDs (Daulatzai, 2017; de la Torre, 2021; Eisenmenger et al., 2023) and considerable overlap between vascular cognitive impairment and AD has been suggested (Duncombe et al., 2017). In the mouse model of chronic cerebral hypoperfusion, it was demonstrated that NOX2-mediated oxidative damage caused cerebral blood flow dysregulation and microvascular inflammation leading to cognitive decline (Alfieri et al., 2022). Importantly, all these disturbances were prevented by the genetic deletion of NOX2, indicating that NOX2 may have a critical role in mediating vascular changes in pathological conditions.

Fibrin co-localizes with amyloid plaques and activates microglial NOX (Ryu et al., 2018). A recent study has demonstrated that fibrin immunotherapy in 5xFAD AD model mice was effective in preventing microglial activation and neuronal loss, as well as reducing key pathological pathways, such as the complement pathway, antigen presentation, cytokine response, lysozyme, and ROS (Ryu et al., 2018). Subsequently, another study (Merlini et al., 2019) showed that fibrin-induced microglial NOX activation led to a significant elimination of dendritic spines, suggesting a potential mechanism behind known dendritic dystrophy in plaque proximity in AD and other neurodegenerative diseases (Herms and Dorostkar, 2016).

Accumulation of hyperphosphorylated tau protein is a major hallmark of AD, FTD, and other tauopathies that has been strongly associated with cognitive decline (Barthélemy et al., 2020; Carroll et al., 2021) and glucose hypometabolism (Arendt et al., 2015). Aggregated tau activates NOX (Esteras et al., 2021) and recent findings (Luengo et al., 2022) have shown that the expression of NOX4 (primarily in neurons) is elevated in the presence of pathological hyperphosphorylated tau in human AD brains and a humanized mouse model of tauopathy. Using knockout or neuronal-targeted knockdown of the Nox4 gene in mice, researchers were able to reduce the levels of pathological hyperphosphorylated tau, prevent brain atrophy and synaptic dysfunction, and ultimately prevent cognitive decline. Another study reported that NOX2 deficiency attenuated cognitive impairment and tau pathology in a APP/PS1 mouse AD model (Gong et al., 2020), confirming that NOX activation promotes tau hyperphosphorylation. These results provide further evidence of the critical role of NOXs in AD and FTD pathogenesis and suggest that NOX may be a promising therapeutic target for the treatment of tauopathies.

### 4.2. Parkinson’s Disease

Main genetic factors for autosomal recessive PD such as PINK1, DJ-1, and SNCA A53T mutations have been shown to directly regulate NOX activity (Belarbi et al., 2017). Animal MTPT-based PD model was shown to recapitulate PD-associated neuroinflammation and oxidative stress paralleled by NOX upregulation, while MTPT-treated mice lacking NOX displayed substantially reduced neuronal loss and oxidative stress (Wu et al., 2003). NOX2 activation has been shown to induce post-translational modification of α-synuclein (Keeney et al., 2022), a key process in PD pathogenesis associated with dopaminergic neuron degeneration (Alafuzoff et al., 2009). In turn, oligomeric α-synuclein has been shown to activate microglia, triggering neuroinflammation (Abramov et al., 2020; Pajares et al., 2020; Zhang et al., 2018) with NOX playing a major role in this process (Belarbi et al., 2017; Hou et al., 2018; Pajares et al., 2020; Wang et al., 2015; Zhang et al., 2005). While most previous studies have focused on the relevance of NOX2 in PD-related microglial activation, a recent study (Keeney et al., 2022) demonstrated that both neuronal and microglial NOX2 are highly active in the substantia nigra under chronic conditions in human idiopathic PD and two animal PD models. Moreover, the authors found that neuronal NOX2 was activated by α-synuclein and had a primary role in the initiation of oxidative stress followed by a delayed activation of microglial NOX2, suggesting a pivotal role of neuronal NOX2 in PD pathogenesis. Other neuronal NOX isoforms also play a role in PD: NOX1 has been shown to be expressed specifically in dopaminergic neurons of PD patients and in PD mouse models where it mediated oxidative stress and neurodegeneration (Choi et al., 2012), and increased NOX4 expression was reported in dopaminergic neurons of PD patients where it correlated with negative clinical outcomes and oxidative stress and neurodegeneration (Zawada et al., 2015).

### 4.3. Acquired epilepsy

Recent clinical reports (Pecorelli et al., 2015; Petrillo et al., 2021) suggested a key role of NOX activity in oxidative stress during human epilepsy. Comprehensive reviews on post-traumatic/stroke epilepsy proposed a critical contribution of NOX activation to pathology (Eastman et al., 2020; Lee et al., 2018), while animal studies pointed to NOX involvement in seizure-induced oxidative stress years back (Di Maio et al., 2011; Jaiswal and Kumar, 2022; Kim et al., 2013; Kovac et al., 2014; Patel et al., 2005; Pestana et al., 2010; Tawfeeq Shekh-Ahmad et al., 2019; Tannich et al., 2020). Increased expression and activity of NOX is associated with brain tissue injury and has been shown to be a major source of ROS contributing to post-TBI epilepsy pathology (Eastman et al., 2020; Pecorelli et al., 2015; Petrillo et al., 2021; Zilberter et al., 2022). Recent studies have also reported increased expression and activity of NOX associated with seizure generation in chemoconvulsant-induced models of human temporal lobe epilepsy (TLE), which suggests a key role of NOX-mediated oxidative stress in epileptogenesis (Jaiswal and Kumar, 2022; Patel et al., 2005; Tawfeeq Shekh-Ahmad et al., 2019; Tannich et al., 2020; Williams et al., 2015). Notably, Shekh-Ahmad et al. (Tawfeeq Shekh-Ahmad et al., 2019) demonstrated in a kainate-induced TLE model that inhibition of NOX abolished seizure-induced toxic consequences in cortical cells and largely diminished epileptiform activity. In another recent study, the authors found that chronic brain NOX2 inhibition reduced the number of seizures in a kainate-induced rat epilepsy model (Singh et al., 2022). *In vitro* studies suggest that NOX activation is a primary source of ROS generation and oxidative stress during seizure-like activity in brain slices and that inhibition of NOX is neuroprotective (Kovac et al., 2014; Williams et al., 2015). We have demonstrated that NOX activation is a trigger of seizure-like events in hippocampal slices (Malkov et al., 2019). This seizure-initiating NOX activation was mediated by NMDAR signaling, and the resulting ROS production correlated with glutamate release, potentially indicating a feedback loop where ictal events could perpetuate themselves by promoting further NMDAR-related excitotoxicity and subsequent NOX hyperactivation. Finally, in the same study, we showed that in vivo NOX inhibition blocked seizures and reduced hyperactivity in several murine seizure models (Malkov et al., 2019).

Epilepsy is also a well-established early co-morbidity of AD (Palop and Mucke, 2009; Vossel et al., 2016). Investigating the acute effects of AD-associated Aβ toxicity on network hyperactivity (Minkeviciene et al., 2009; Zilberter et al., 2013), we found that intracerebroventricular injection of Aβ resulted in long-lasting (at least days) network hyperactivity (seen as increased interictal spike frequency as well as the appearance of pathological high-frequency oscillations) and glucose hypometabolism. These effects were completely prevented by NOX2 inhibition (Malkov et al., 2020), suggesting that NOX2-derived oxidative stress is directly responsible for AD-related epileptiform activity.

### 4.4. Amyotrophic lateral sclerosis

NOX4 gene has been linked to sporadic ALS in a GWAS study (Dunckley et al., 2007). NOX2 hyperactivation has been reported to be negatively predictive of survival in ALS patients independent from other factors (Marrali et al., 2014), and NOX2 expression was reported to be increased in microglia of ALS patients as well as in spinal cords of three different mouse ALS models by up to 10–60 times (Apolloni et al., 2013a, 2013b; Seredenina et al., 2016; Wu et al., 2006). Wild-type and mutant forms of transactive response DNA-binding protein-43 (TDP-43) implicated in both ALS and FTD were shown to induce neuronal toxicity via NOX2-mediated microglial reactivity (W. Zhao et al., 2015). NOX2 activation has also been shown to be directly mediated by ALS-related glial superoxide dismutase 1 (SOD1) variant (Harraz et al., 2008). NOX inhibition slowed neurodegeneration and extended survival in ALS model mice (Marden et al., 2007; Wu et al., 2006), an effect replicated in another study on treating ALS mice with NOX inhibitor apocynin (Harraz et al., 2008). However, these results have been contradicted by Seredinina et al. (Seredenina et al., 2016) who showed that although NOX2 expression is increased in SOD1-G93A mice, its global deletion did not extend their survival. Another study (Trumbull et al., 2012) reported that while NOX blockade by non-specific NOX antagonist diapocynin (apocynin derivative) was protective in a motor neuron-SOD1 mutant microglia co-culture, diapocynin treatment in SOD1 mice did not extend lifespan. The reason for such discrepancies is unclear but could be attributed to variability due to mouse background strains in NOX knockout studies, while NOX inhibition treatments likely proved variable due to poor CNS penetrance and specificity of apocynin and its derivatives. Nevertheless, available evidence points to NOX involvement in ALS pathology and more research using novel specific and brain-available NOX inhibitors as well as microglia-specific NOX deletion is needed.

### 4.5. Multiple sclerosis

While reports of energy deficiency and resulting tissue damage in MS largely focused on the role of mitochondrial injury (Dutta et al., 2006; Lu et al., 2000; Mahad et al., 2008), microglial NOX was directly implicated as the source of the oxidative burst initiating and driving these pathologies in active and slowly expanding lesions (Fischer et al., 2012; Panday et al., 2015) as well as during the remission phase (Radbruch et al., 2016). NOX is abundantly expressed in pre-lesion microglial clusters in brains of MS patients (van Horssen et al., 2012), and increased NOX5 and decreased NOX4 concentrations were found in serum samples from relapsing remitting MS patients (Doğan and Yildiz, 2019). Brain NOX2 expression is also increased in a experimental autoimmune encephalomyelitis MS model (Zarruk et al., 2015), and its activation was shown to be key to the experimental autoimmune encephalomyelitis (EAE) disease pathogenesis (Hu et al., 2021; Ravelli et al., 2019) as well as to the failure of hippocampal long-term synaptic plasticity behind cognitive and behavioral alterations (Di Filippo et al., 2016), suggesting that NOX activation may be responsible for both tissue damage as well as for synaptic and cognitive deficits associated with MS. Confirming these results, NOX inhibition by apocynin dramatically reduced the symptoms in the EAE model as well as abated demylienation and peripheral macrophage infiltration (Choi et al., 2015). In summary, the multifaceted involvement of NOX in MS pathogenesis underscores its potential as a therapeutic target for mitigating tissue damage, synaptic dysfunction, and cognitive deficits associated with the disease.

### 4.6. Acute neurological disorders

Finally, a number of recent studies have shown NOX to be a major source of oxidative stress responsible for neuronal death in acute neurological disorders such as stroke, TBI, and hypoglycemia-related neuronal injury (Abramov et al., 2020; Barua et al., 2019; Begum et al., 2022; Hou et al., 2020; Lee et al., 2021; Tang et al., 2011; Tarafdar and Pula, 2018; Wang et al., 2013).

## 5. The role of NOX in neuroinflammation

Neuroinflammation is another early hallmark of major neurodegenerative disorders, implicated in major NDDs including AD (Ahmad et al., 2022; Dhapola et al., 2021; Uddin et al., 2020), PD (McGeer et al., 1988a, 1988b; Orr et al., 2005), FTD ((Bright et al., 2019; Heneka et al., 2014), acquired epilepsy ((Pauletti et al., 2019; Vezzani et al., 2019), ALS (Barbeito et al., 2010; Calvo et al., 2010; Martínez et al., 2020; McCombe and Henderson, 2011; McGeer and McGeer, 2002; Zhao et al., 2013), and MS (Hauser and Cree, 2020; Kamma et al., 2022). Neuroinflammation represents a complex brain defense mechanism against diverse pathogens and injuries. The key cell type in the process, microglia constitute approximately 5–12% of the total brain cell population (Kwon and Koh, 2020) but can proliferate (Vela et al., 2002). Upon activation, microglia release a variety of proinflammatory cytokines and chemokines to recruit immune cells and modulate the brain’s immune response. Protective in the healthy brain, prolonged inflammatory activation of microglia and astrocytes is thought to contribute to the progression of NDDs (Chitnis and Weiner, 2017). This occurs through various mechanisms, including the microglia-mediated synaptic phagocytosis, upregulation of kinases leading to tau hyperphosphorylation, β-amyloid production and aggregation, and activation of the NLRP3 pathway (discussed in (Kinney et al., 2018)). The two largest genetic risk factors for sporadic AD, apolipoprotein E ε4 (APOE4) and variants of triggering receptor expressed on myeloid cells 2 (TREM2) (Wolfe et al., 2018), are implicated in microglial activation and neuroinflammation (Andronie-Cioara et al., 2023; Fernandez et al., 2019; Ferrari-Souza et al., 2023; Guerreiro et al., 2013; Keren-Shaul et al., 2017; Koutsodendris et al., 2023; Yuan et al., 2020; Zalocusky et al., 2021). Activated microglia are also capable of generating ROS via a NOX-generated “oxidative burst” in response to various stimuli, including aggregated proteins such as Aβ in Alzheimer’s disease and α-synuclein in Parkinson’s disease (Simpson and Oliver, 2020; Tarafdar and Pula, 2018). Like phagocytes, microglia predominantly use NOX for ROS production, with the NOX2 isoform being the most abundantly expressed in human microglia (Hou et al., 2020; Sorce et al., 2017). In turn, NOX activity has been shown to be pro-inflammatory (Hou et al., 2020). Recent studies have shown that microglial NOX can be activated by fibrin (Merlini et al., 2019; Ryu et al., 2018), an insoluble product of fibrinogen that accumulates in the brain as a result of blood-brain barrier dysfunction in neurodegenerative diseases such as MS and AD (Z. Zhao et al., 2015). Fibrin-targeting immunotherapy has been shown to reduce neuroinflammation and neurodegeneration in animal models of these diseases (Ryu et al., 2018), highlighting the contribution of activated microglia to oxidative stress and inflammation during NDD pathogenesis. Ultimately, NOX-mediated neuroinflammatory processes contribute to the state of initiating oxidative stress that triggers the onset and progression of NDDs.

## 6. Approaches to counteracting NOX-mediated iOS in humans

Our data has revealed that even the most potent antioxidants, when applied directly to active brain tissue, proved ineffective in countering the rapid accumulation of ROS and their associated effects caused by NOX hyperactivation (Malkov et al., 2018). This finding may shed light on the apparent discrepancy of repeated failures of antioxidant trials for NDDs (Angelova and Abramov, 2018; Butterfield and Halliwell, 2019; Chopade et al., 2023; Ienco et al., 2011; Jurcau, 2021; Kim et al., 2015; Kumar and Singh, 2015; Liu et al., 2017; Mecocci and Polidori, 2012; Perez Ortiz and Swerdlow, 2019; Pohl and Kong Thoo Lin, 2018; Singh et al., 2019; Wang et al., 2019) and implies that a more promising treatment approach lies in preventing ROS accumulation by directly inhibiting NOX hyperactivity. However, NOX enzymes generate superoxide in phagocytes which play critical roles in human immune response (Bedard and Krause, 2007), making non-selective inhibition of NOXs a non-starter in any NOX-targeting NDD treatment. To avoid harmful side effects, only selective inhibition of NOX isoforms specific to the brain should be considered. Targeting brain NOX activity without any off-target systemic effects has been impossible until recently due to the lack of isoform-specific and brain-penetrant antagonists. Several NOX inhibitors have been analyzed in recent comprehensive reviews (Augsburger et al., 2019; Begum et al., 2022; Chocry and Leloup, 2020; Elbatreek et al., 2021) but only a few selective antagonists for NOX2 and NOX4 isoforms have been synthesized, and only one (GKT137831, a specific inhibitor of NOX1 and NOX4) is in human clinical trials (phase 2) for pulmonary fibrosis and cirrhosis (Begum et al., 2022). Several other promising inhibitors have been recently developed, such as NOS31 for NOX1, GLX7013114 for NOX4, and GSK2795039 for NOX2 recently modified and improved (Hirano et al., 2015; Mason et al., 2023). It is also important to mention the very recent study of Juric et al. who reported the development of novel brain-permeable Nox2 inhibitors (Juric et al., 2023). Altogether, as NOX isoforms have been well identified and studied, the design and testing of efficient, selective, and brain-available inhibitors is hopefully a matter of the near future.

## 7. Conclusion

Our review highlights the essential role of oxidative stress in neurodegenerative diseases, emphasizing its contribution as a major initiating factor rather than a mere parallel pathology. Recent evidence has pointed to oxidative stress as the primary culprit behind the reduced glucose metabolism in the brain, another hallmark of NDD prodromal stages. Notably, hyperactivation of NOX enzymes has emerged as a significant source of this initial oxidative stress, making inhibiting NOX activity a potentially effective alternative to failed general antioxidant treatments and therefore a promising strategy for preventing the onset of major NDDs. Based on available data, we postulate a general hypothesis of sporadic NDD pathogenesis (Fig. 1): various disease-specific risk factors, both genetic and environmental, trigger initiating oxidative stress and neuroinflammation, with NOX activation playing a major role. Initiating oxidative stress induces pathological changes in brain glucose utilization, resulting in network dysfunction and multiple neurodegenerative cascades. This is a triggering phase of disease progress, where the convergence of oxidative stress, neuroinflammation, and NOX hyperactivation sets in motion a cascade of events leading to chronic oxidative stress-neurodegeneration cycles, perpetuating the pathogenesis of sporadic NDDs. The identification and characterization of NOX isoforms enabled the development of efficient, selective, and brain-penetrant NOX inhibitors, bringing us closer to a viable therapeutic approach for sporadic NDDs. Collectively, our findings emphasize the importance of targeting NOX activity as a treatment strategy for neurodegenerative diseases.

## Data Availability

No data was used for the research described in the article.
